# Prophylactic Repetitive Treatment with the Herbal Medicine Kei-kyoh-zoh-soh-oh-shin-bu-toh Attenuates Oxaliplatin-Induced Mechanical Allodynia by Decreasing Spinal Astrocytes

**DOI:** 10.1155/2019/4029694

**Published:** 2019-04-17

**Authors:** Tsugunobu Andoh, Daisuke Fukutomi, Daisuke Uta, Yasushi Kuraishi

**Affiliations:** Department of Applied Pharmacology, Graduate School of Medicine and Pharmaceutical Sciences, University of Toyama, Toyama 930-0194, Japan

## Abstract

Chemotherapeutic drugs typically induce peripheral neuropathy, which is a major dose-limiting side effect of these drugs and is difficult to manage. In this study, we examined whether the traditional herbal formulation Kei-kyoh-zoh-soh-oh-shin-bu-toh (KSOT) could relieve the mechanical allodynia induced by chemotherapeutic drugs (oxaliplatin, paclitaxel, vincristine, and bortezomib) in mice. A single intraperitoneal injection of oxaliplatin, paclitaxel, vincristine, and bortezomib was used to induce mechanical allodynia, which peaked on days 10, 14, 14, and 12 after the injection, respectively. A single oral administration of KSOT did not inhibit mechanical allodynia after any of the treatments. However, prophylactic repetitive oral administrations of KSOT inhibited the exacerbation of mechanical allodynia induced by oxaliplatin but were not effective for allodynia induced by the other drugs. A single intraperitoneal injection of oxaliplatin did not alter the mRNA expression of the NMDA receptor NR2B in the spinal cord and that of neuregulin-1 in the sciatic nerve. In addition, the number of microglia in spinal dorsal horn did not increase in oxaliplatin-treated mice. However, the number of reactivated astrocytes in the spinal dorsal horn increased, which could be inhibited by repetitive administration of KSOT. These results suggest that prophylactic repetitive treatment of KSOT attenuates oxaliplatin-induced mechanical allodynia by decreasing the number of spinal astrocytes.

## 1. Introduction

Most cancer patients receive anticancer drugs such as the platinum-based drug oxaliplatin, the taxane paclitaxel, the vinca alkaloid vincristine, and the proteasome holoenzyme inhibitor bortezomib. However, these anticancer drugs induce peripheral neuropathy, which is a major dose-limiting side effect and is characterized by dysesthesias, including allodynia and cold hypersensitivity [1–4]. Peripheral neuropathy hampers the quality of life in patients and interferes with the chemotherapy. Therefore, controlling the peripheral neuropathy is very important for cancer patients who receive anticancer drugs. Although vitamin E, glutathione, gabapentin, duloxetine, etc. are effective for managing chemotherapy-induced peripheral neuropathy, their efficacy depends on the chemotherapeutic agents used [3, 5].

Kei-kyoh-zoh-soh-oh-shin-bu-toh (KSOT) is a traditional herbal formulation consisting of seven herbal medicines: Cinnamomi cortex (*Cinnamomum cassia* Blume: “keihi”), Zingiberis rhizoma (*Zingiber officinale* Roscoe: “shokyoh”), Ziziphi fructus (*Ziziphus jujuba* Miller var.* inermis* Rehder: “taisoh”), Glycyrrhizae radix (*Glycyrrhiza uralensis* Fischer: “kanzoh”), Ephedrae herba (*Ephedra sinica* Stapf: “maoh”), Asiasari radix (*Asiasarum sieboldii* F. Maekawa: “saisin” ), and Aconiti Tuber (*Aconitum carmichaeli* Debeaux: “bushi”). KSOT is effective in treating chronic pains, such as neuralgia, rheumatalgia, and lower back pain in patients [6]. In rodents (rats and mice), KSOT inhibits hyperalgesia induced by repeated cold stress or adjuvant prepared with heat-killed* Mycobacterium tuberculosis* [7, 8]. However, it is unknown whether KSOT is effective against chemotherapy-induced mechanical allodynia. Therefore, in this study, we investigated the effects of KSOT on mechanical allodynia induced by chemotherapeutic drugs (oxaliplatin, paclitaxel, vincristine, and bortezomib) in mice. In addition, we also investigated the mechanisms underlying the antiallodynic action of KSOT.

## 2. Materials and Methods

### 2.1. Animals

Male C57BL/6 mice (Japan SLC Ltd., Hamamatsu, Japan) (6 weeks of age at the start of experiments) were used. They were housed under controlled temperature (21-23°C) and humidity (45%-46%). The room was lighted from 0700 h to 1900 h. Food and water were available* ad libitum*. The study was conducted with the approval of the Committee for Animal Experiments at the University of Toyama.

### 2.2. Drugs

Oxaliplatin (Wako Pure Chemical Industries, Osaka, Japan) was dissolved in a vehicle (5% glucose) and administered intraperitoneally at a dose of 3 mg/kg [9]. Paclitaxel (Sigma-Aldrich) was dissolved in a vehicle (saline containing 10% (v/v) Cremophor (Sigma-Aldrich) and 10% (v/v) ethanol) and administered intraperitoneally at a dose of 5 mg/kg [9]. Vincristine sulfate (Sigma-Aldrich, St. Louis, MO, USA) was dissolved in a vehicle (saline) and administered intraperitoneally at a dose of 0.1 mg/kg [9]. Bortezomib (Toronto Research Chemicals, Ltd., Toronto, Canada) was dissolved in a vehicle (saline containing 0.03% (v/v) mannitol (Sigma-Aldrich)) and administered intravenously at a dose of 0.3 mg/kg [10].* Kei-kyoh-zoh-soh-oh-shin-bu-toh (KSOT, TJ-8023, Lot. 938023002P0), as a powder spray-dried after extracting crude drugs (Table 1) with boiling water, was obtained from Tumura & Co. Ltd. (Tokyo, Japan) [8]. KSOT was vacuum packaged and stored at -80*°*C until use. KSOT was dissolved in a vehicle (5% gum Arabic) and administered orally*. The chemotherapeutic drugs and KSOT were administered intraperitoneally and orally, respectively, at a volume of 0.1 ml/10g of body weight. KSOT was administered orally once daily, starting the day after chemotherapeutic drug injection. In a part of the experiment, a single oral administration of KSOT was performed at the peak of mechanical allodynia induced by chemotherapeutic drug injection.

### 2.3. Behavioral Experiments

Mice were placed individually in a plastic cage (11 cm ×18 cm × 15 cm) with a wire mesh bottom and allowed to acclimatize for at least 30 min. Mechanical allodynia in the hind paw was evaluated with a fine von Frey filament (a bending force of 0.69 mN <innocuous stimulation>, North Coast Medical Inc., Morgan Hill, CA, USA) [9]. The von Frey filament was pressed perpendicularly against the central part of the plantar hind paw and was held there for 1–3 s by slight buckling. Responses to the stimulus were scored as follows: no reaction (0), lifting of the hind paw (1), and licking and flinching of the hind paw (2). A stimulation of same intensity was applied three times to each hind paw at intervals of several seconds and the average value of six trials was used as the response score (the maximum score being 2). Cold dysesthesia was evaluated using acetone [11]. Acetone (10 *μ*l) was applied to the plantar skin. Aversive responses during the 10-s period following acetone stimulation were scored as follows: no response (0), lifting of the hind paw (1), and flinching or licking of the hind paw (2). Naïve mice showed a transient escape response immediately after acetone application, and this response was disregarded. Acetone was applied three times alternately to each hind paw at intervals of more than 20 s, and the average of six trials served as the aversive response score.

### 2.4. Western Blotting

On day 10 after oxaliplatin injection, mice were anesthetized with pentobarbital sodium (70 mg/kg, intraperitoneal) and then transcardially perfused with phosphate buffered saline (PBS). The spinal cord was removed and the proteins were extracted using lysis buffer (20 mM Tris-HCl [pH 7.5], 137 mM NaCl, 1% NP-40, 10% glycerol, 1 mM phenylmethylsulfonyl fluoride, 10 *μ*g/mL aprotinin, and 1 *μ*g/mL leupeptin). Protein concentration was determined by the Bradford assay (Bio-Rad, Hercules, CA, USA). The proteins were separated by SDS-PAGE and transferred to a polyvinylidene difluoride membrane (Bio-Rad). The membrane was pretreated with 5% skim milk (Wako Pure Chemical Industries) in Tris-buffered saline containing 0.1% Tween 20 (TBS-T) for 1 h and then incubated with a mouse anti-NR2B monoclonal antibody (1:1,000, BD biosciences, Danvers, MA, USA), rabbit anti-neuregulin-1 (NRG1) Type III polyclonal antibody (1:2,500, abcam, MA, USA), or rabbit anti-GAPDH polyclonal antibody (1:10,000, IMGENEX, San Diego, CA, USA) at 4°C overnight. This was followed by incubation with horseradish peroxidase-conjugated anti-mouse or anti-rabbit IgG (1:2000, GE Healthcare Bio-Sciences Co., Piscataway, NJ, USA) for 1 h at room temperature. The signals were visualized by chemiluminescence reaction (GE Healthcare) using X-ray films and analyzed using the NIH program, Image J. The signal intensity was normalized to that of GAPDH.

### 2.5. Immunohistochemistry

On day 10 after oxaliplatin injection, mice were anesthetized with pentobarbital sodium (70 mg/kg, intraperitoneal) and then transcardially perfused with 4% paraformaldehyde (PFA), following treatment with PBS. The spinal cord was removed and soaked in 30% sucrose at 4°C for a couple of days, following the treatment with 4% PFA for 1 day. The tissue was embedded in OCT compound and was stored at -80°C until use. Sections (30 *μ*m) of the spinal cord were prepared using a cryostat (Leica CM 3050S IV, Leica Biosystems, Wetzlar, Germany) and were preserved in PBS at 4°C. After soaking the sections with PBS containing 0.2% Triton X-100 (PBST), blocking was performed with PBST containing 1.5% fetal bovine serum (FBS) at room temperature for 30 min. The sections were then incubated overnight with rat antiglial fibrillary acidic protein (GFAP) polyclonal antibody (1:2,000, Invitrogen, Inc., Carlsbad, CA, USA) or rabbit anti-Iba1 polyclonal antibody (1:2,000, Wako Pure Chemical Industries) at 4°C. After washing with PBST, the sections were incubated with Cy3-labeled Goat anti-rat IgG (1:1,000, KPL, Inc., Gaithersburg, MD, USA) or Alexa 488-labeled donkey anti-rabbit IgG antibody (1:1,000, Life technologies, Eugene, OR, USA) at room temperature for 1 h. The sections were rinsed with PBST and were mounted using a mounting medium with DAPI (VECTOR LABORATORIES, Inc., Burlingame, CA, USA). Fluorescence signals were observed using a confocal laser-scanning microscope (LSM780, Carl Zeiss, Oberkochen, Germany).

### 2.6. Statistical Analyses

Statistical analyses were performed using the SigmaPlot software version 11 (Systat Software, Ltd., Chicago, IL, USA). Data are represented as mean ± standard error of the mean. Statistical significance was evaluated by two-way repeated measures analysis of variance (ANOVA) or one-way ANOVA, followed by post hoc Holm-Šidák multiple comparisons test.* P* < 0.05 indicated significance.

## 3. Results

### 3.1. Oxaliplatin-, Paclitaxel-, Vincristine- or Bortezomib-Induced Mechanical Allodynia in Mice

**Figure 1:**
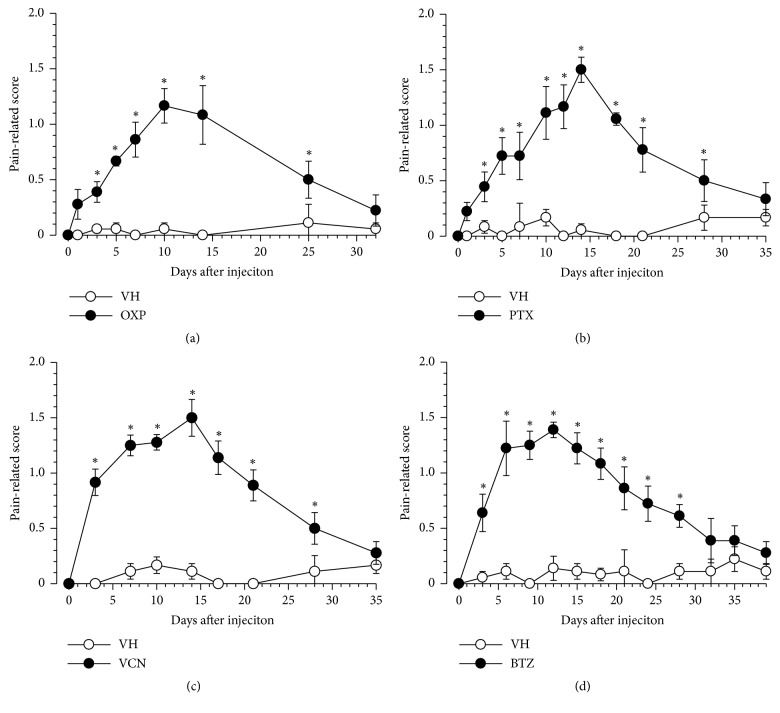
Development of mechanical allodynia induced by a single injection of chemotherapeutic agents. Mice were given a single injection of (a) oxaliplatin (OXP, 3 mg/kg, i.p.), (b) paclitaxel (PTX, 5 mg/kg, i.p.), (c) vincristine (VCN, 0.1 mg/kg, i.p.), (d) bortezomib (BTZ, 0.3 mg/kg, i.v.) or the corresponding vehicle (VH) on day 0. Mechanical allodynia was evaluated using a fine von Frey filament (0.69 mN strength). Data are presented as mean ± standard errors of the mean (n = 6). *∗P* < 0.05 vs. VH (Holm-Šidák multiple comparisons). i.p.: intraperitoneal injection, i.v.: intravenous injection.

A single intraperitoneal injection of oxaliplatin (0.3 mg/kg, i.p.) (Figure 1(a)), paclitaxel (5 mg/kg, i.p.) (Figure 1(b)), vincristine (0.1 mg/kg, i.p.) (Figure 1(c)), or bortezomib (0.3 mg/k, i.p.) (Figure 1(d)) was used to induce mechanical allodynia, which peaked on days 10, 14, 14 or 12 after the injection, respectively (Figure 1). Peak allodynia was not prominently different between these chemotherapeutic agents. Mechanical allodynia almost subsided on days 30, 35, 35 or 32 after the injection of oxaliplatin, paclitaxel, vincristine, or bortezomib, respectively (Figure 1). However, each vehicle did not induce mechanical allodynia (Figure 1). The time-course of mechanical allodynia induced by these chemotherapeutic drugs used in this study is similar to our previous reports [9, 10].

### 3.2. Effect of Single or Repetitive Prophylactic Administration of KSOT on Mechanical Allodynia Induced by Chemotherapeutic Agents

**Figure 2:**
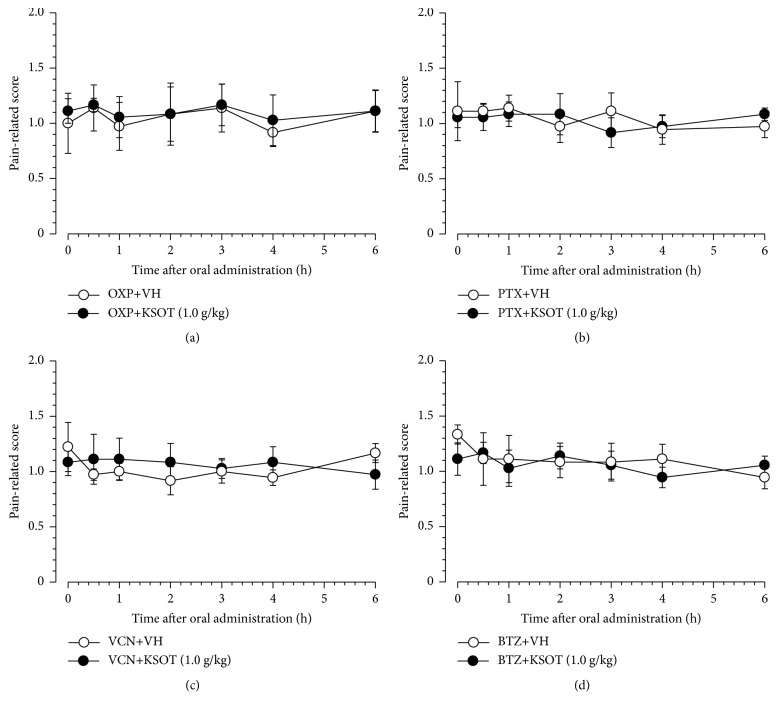
Effect of a single oral administration of Kei-kyoh-zoh-soh-oh-shin-bu-toh (KSOT) on the mechanical allodynia induced by chemotherapeutic agents. Mice were given a single injection of (a) oxaliplatin (OXP, 3 mg/kg, i.p.), (b) paclitaxel (PTX, 5 mg/kg, i.p.), (c) vincristine (VCN, 0.1 mg/kg, i.p.), or (d) bortezomib (BTZ, 0.3 mg/kg, i.v.) on day 0. KSOT or the vehicle (VH) was orally administered on day 10 (for OXP-treated mice), day 14 (for PTX- or VCN-treated mice), or day 12 (for BTZ-treated mice) after the injection. Mechanical allodynia was evaluated using a fine von Frey filament (0.69 mN strength). Data are presented as mean ± standard errors of the mean (n = 6). i.p.: intraperitoneal injection, i.v.: intravenous injection.

**Figure 3:**
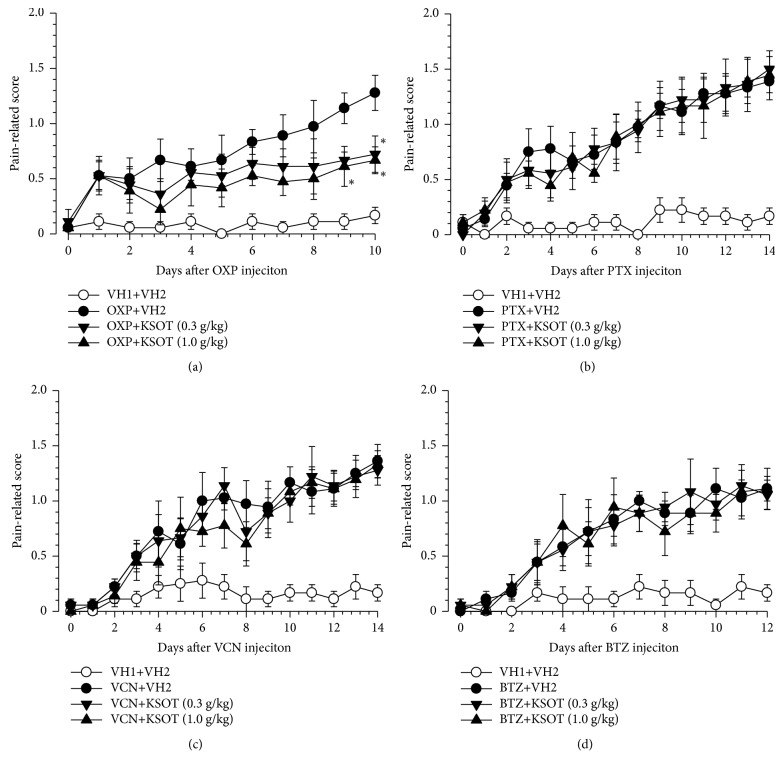
Effect of prophylactic repetitive oral administration of Kei-kyoh-zoh-soh-oh-shin-bu-toh (KSOT) on the mechanical allodynia induced by chemotherapeutic agents. Mice were given a single injection of (a) oxaliplatin (OXP, 3 mg/kg, i.p.), (b) paclitaxel (PTX, 5 mg/kg, i.p.), (c) vincristine (VCN, 0.1 mg/kg, i.p.), (d) bortezomib (BTZ, 0.3 mg/kg, i.v.) or the corresponding vehicles (VH1) on day 0. KSOT or the vehicle (VH2) orally administered once daily from the day after a single injection of chemotherapeutic agent or the corresponding vehicle (VH1). Mechanical allodynia was evaluated using a fine von Frey filament (0.69 mN strength). Data are presented as mean ± standard errors of the mean (n = 6). *∗P* < 0.05 vs. OXP + VH2 (Holm-Šidák multiple comparisons). i.p.: intraperitoneal injection, i.v.: intravenous injection.

To evaluate acute effect, KSOT was given once at the peak of mechanical allodynia induced by each chemotherapeutic drugs. A single administration of KSOT (1.0 g/kg) did not inhibit mechanical allodynia induced by oxaliplatin, paclitaxel, vincristine, or bortezomib at least for the period evaluated (Figure 2). As next experiment, prophylactic repetitive oral administration of KSOT was evaluated. Prophylactic repetitive oral administration of KSOT (0.3 and 1.0 g/kg) significantly inhibited the exacerbation of mechanical allodynia from day 9 after oxaliplatin injection, compared to that observed in the vehicle-treated group. Two-way RM-ANOVA of data from day 1 to day 10 demonstrated a significant main effect of treatment [*F*_(2,10)_= 4.424,* P*<0.05] (Figure 3(a)). However, KOST (0.3 and 1.0 g/kg) did not affect the mechanical allodynia induced by paclitaxel, vincristine, or bortezomib (Figures 3(b), 3(c), and 3(d)). Prophylactic repetitive oral administration of KSOT (0.3 and 1.0 g/kg) did not induce abnormal behavior at least during the administration period.

### 3.3. Effect of Prophylactic Repetitive Administration of KSOT on Cold Dysesthesia Induced by Oxaliplatin

**Figure 4:**
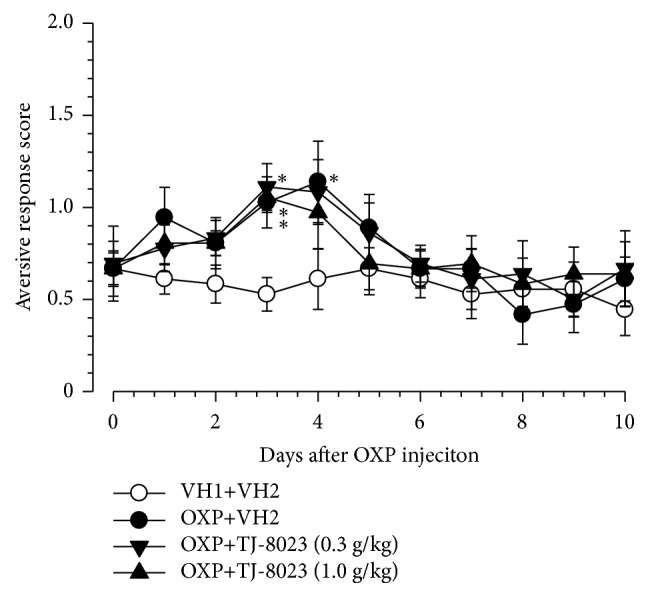
Effect of prophylactic repetitive oral administration of Kei-kyoh-zoh-soh-oh-shin-bu-toh (KSOT) on cold dysesthesia induced by oxaliplatin. Mice were given a single injection of oxaliplatin (OXP, 3 mg/kg, i.p.) or the corresponding vehicle (VH1) on day 0. KSOT or the vehicle (VH2) was orally administered once daily, starting the day after a single injection of oxaliplatin or the corresponding vehicle (VH1). Cold dysesthesia was evaluated using acetone test. Data are presented as mean ± standard errors of the mean (n = 6). *∗P* < 0.05 vs. VH1 + VH2 (Holm-Šidák multiple comparisons). i.p.: intraperitoneal injection.

Cold dysesthesia is a characteristic symptom of oxaliplatin-induced peripheral neuropathy in humans [2] and animals [9, 11, 12]. A single injection of oxaliplatin (0.3 mg/kg, i.p.) induced cold dysesthesia, which peaked on day 3-4 after the injection (Figure 4), and almost subsided by day 6 after the injection (Figure 3). Prophylactic repetitive oral administration of KSOT (0.3 and 1.0 g/kg) did not inhibit the exacerbation of cold dysesthesia (Figure 4).

### 3.4. Effect of Prophylactic Repetitive Administration of KSOT on the Expression of NR2B and NRG1 in Oxaliplatin-Treated Mice

**Figure 5:**
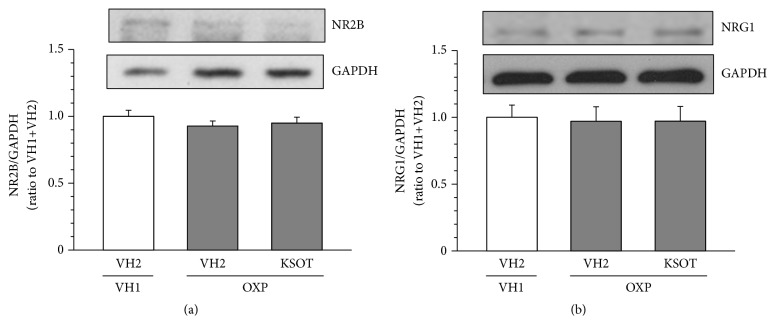
Effect of prophylactic repetitive oral administration of Kei-kyoh-zoh-soh-oh-shin-bu-toh (KSOT) on the expression of NMDA receptor 2B (NR2B) and neuregulin-1 (NRG1) in oxaliplatin-treated mice. Mice were given a single injection of oxaliplatin (OXP, 3 mg/kg, i.p.) or the corresponding vehicle (VH1) on day 0. KSOT or the vehicle (VH2) was orally administered once daily, starting the day after a single injection of OXP or the corresponding vehicle (VH1). The spinal cord for NR2B (a) and the sciatic nerve for NRG1 (b) were isolated on day 10 after OXP injection and the proteins were extracted for Western blotting. The expression level of NR2B or NRG1 in each sample was normalized to that of glyceraldehyde-3-phosphate dehydrogenase (GAPDH) and further normalized to its expression level of the vehicle control (VH1+VH2). (a) A typical example of the expression levels of NR2B or NRG1 and GAPDH. (b) The expression level of NR2B or NRG1, normalized with that of GAPDH. Data are presented as mean ± standard errors of the mean (n = 6 for NR2B; n = 3 for NRG1).

In rats treated with oxaliplatin (4 mg/kg) twice a week for 4 weeks, the expression of NR2B in spinal cord increased [13] and that of NRG1 in sciatic nerve are decreased [14]. In mice on day 10 after a single injection of oxaliplatin (3 mg/kg) in this study, the expression of NR2B in spinal cord and NRG1 in sciatic nerve did not increase, compared with vehicle-treated mice (Figure 5). In addition, prophylactic repetitive oral administration of KSOT (1.0 g/kg) did not affect the expression of NR2B in the spinal cord and NRG1 in sciatic nerve of oxaliplatin-treated mice, compared with repetitive vehicle-administered mice (Figure 5).

### 3.5. Effect of Prophylactic Repetitive Administration of KSOT on Distribution of Microglia and Astrocytes in the Spinal Dorsal Horn of Oxaliplatin-Treated Mice

**Figure 6:**
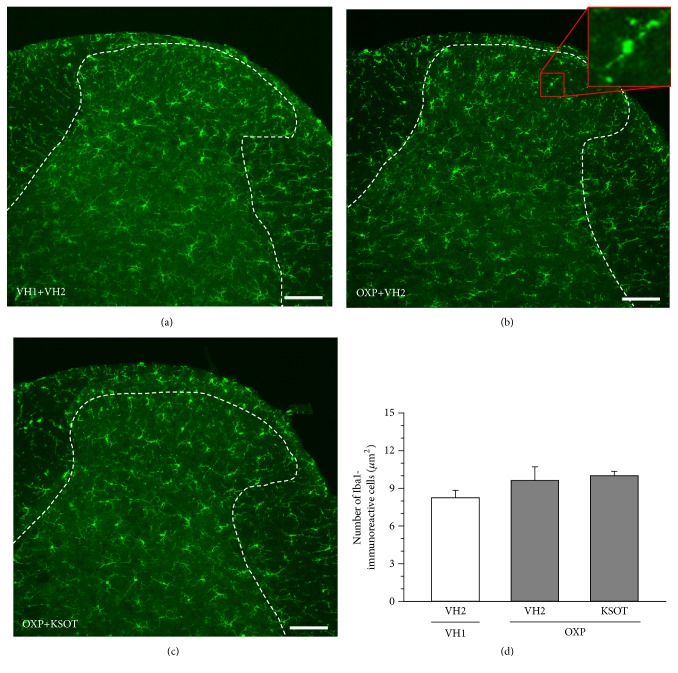
Distribution of microglia in the spinal dorsal horn of oxaliplatin-treated mice and the effect of Kei-kyoh-zoh-soh-oh-shin-bu-toh (KSOT). Mice were given a single injection of oxaliplatin (OXP, 3 mg/kg, i.p.) or the corresponding vehicles (VH1) on day 0. KSOT or the vehicle (VH2) was orally administered once daily, starting the day after a single injection of OXP or the corresponding vehicle (VH1). The spinal cords were isolated on day 10 after OXP injection. Typical examples of the distribution of Iba-1-immunoreactive microglia in mouse spinal dorsal horn: (a) VH1+VH2, (b) OXP + VH2, and (c) OXP+ KSOT. Scale bar = 100 *μ*m. (d) The number of Iba-1-immunoreactive microglia. Data are presented as mean ± standard errors of the mean (n = 4). i.p.: intraperitoneal injection.

**Figure 7:**
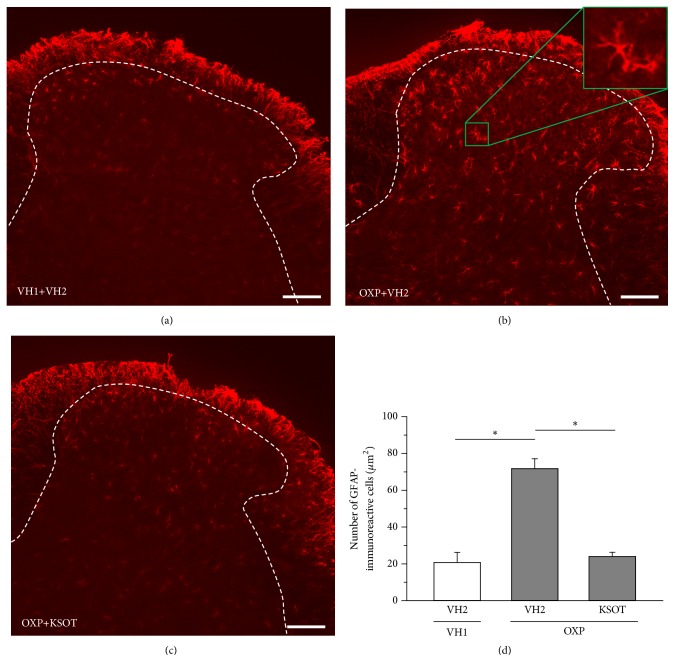
Distribution of astrocytes in the spinal dorsal horn of oxaliplatin-treated mice and the effect of Kei-kyoh-zoh-soh-oh-shin-bu-toh (KSOT). Mice were given a single injection of oxaliplatin (OXP, 3 mg/kg, i.p.) or the corresponding vehicles (VH1) on day 0. KSOT or the vehicle (VH2) was orally administered once daily, starting from the day after a single injection of OXP or the corresponding vehicle (VH1). The spinal cords were isolated on day 10 after OXP injection. Typical examples of the distribution of GFAP-immunoreactive astrocytes in mouse spinal dorsal horn: (a) VH1+VH2, (b) OXP + VH2, and (c) OXP+ KSOT. Scale bar = 100 *μ*m. (d) The number of GFAP-immunoreactive astrocytes. Data are presented as mean ± standard errors of the mean (n = 4). *∗P* < 0.05 (Holm-Šidák multiple comparisons). i.p.: intraperitoneal injection.

Spinal microglia and astrocytes are reactivated by nerve injury [15]. In this study, the reactivated microglia and astrocytes were evaluated using their histopathology. Reactivated microglia is reported to be oversized, bloated, and star-shaped cells [16]. As for the characteristics of reactivated astrocytes, hypertrophy of the cell soma and processes, as well as upregulation of GFAP, is observed [17]. A single injection of oxaliplatin did not increase the number of reactivated microglia in spinal dorsal horn of mice 10 days after the injection (Figures 6(a), 6(b), and 6(d)). In addition, prophylactic repetitive oral administration of KSOT (1.0 g/kg) did not affect the distribution of reactivated microglia in spinal dorsal horn of oxaliplatin-treated mice (Figures 6(b), 6(c), and 6(d)). On the other hand, a single injection of oxaliplatin increased the number of reactivated astrocytes in spinal dorsal horn of mice 10 days after the injection (Figures 7(a), 7(b), and 7(d)). Prophylactic repetitive oral administration of KSOT (1.0 g/kg) inhibited the increase in the number of reactivated astrocytes in spinal dorsal horn of oxaliplatin-treated mice (Figures 7(b), 7(c), and 7(d)).

## 4. Discussion

Peripheral neuropathy is one of the side effects of anticancer drugs (e.g., oxaliplatin, paclitaxel, vincristine, and bortezomib) in humans [1–4]. In mice, these drugs (oxaliplatin, paclitaxel, vincristine, and bortezomib) are also known to induce mechanical allodynia [9, 10] and partly cold dysesthesia (oxaliplatin) [11]. In this study, a single oral administration of KSOT did not affect the mechanical allodynia induced by the studied chemotherapeutic agents. One the other hand, prophylactic repetitive oral administration of KSOT inhibited the exacerbation of mechanical allodynia induced by oxaliplatin but was not effective in the case of paclitaxel, vincristine, and bortezomib. In addition, repetitive KSOT did not affect oxaliplatin-induced cold dysesthesia. These results suggest that prophylactic repetitive oral administration of KSOT is effective for oxaliplatin-induced mechanical allodynia.

The descending noradrenergic and serotonergic systems play an important role in the endogenous pain inhibitory system [18]. A single administration of KSOT attenuates repeated cold stress (RCS)-induced hyperalgesia through the activation of descending serotonergic, but not noradrenergic, system in rats [8]. Another traditional herbal formulation goshajinkigan also inhibits oxaliplatin-induced mechanical allodynia through the activation of descending serotonergic and noradrenergic system in mice [19]. In addition, gabapentin suppresses bortezomib-induced mechanical allodynia through the activation of descending noradrenergic, but not serotonergic, system in mice [10]. In this study, a single administration of KSOT did not inhibit oxaliplatin-induced mechanical allodynia. Chemotherapeutic agents induce neuropathic pain, whereas RCS does not. Furthermore, the above findings suggest that the activation of descending noradrenergic, but not serotonergic, system regulates chemotherapeutic agent-induced mechanical allodynia. As KSOT activates the descending serotonergic system, the role of descending pain inhibitory system on the inhibition of the exacerbation of oxaliplatin-induced mechanical allodynia by repetitive KSOT administration is small.

It is well known that NMDA glutamate receptors, especially NR2B subunit, expressed after synapse in the spinal dorsal horn is involved in neuropathic pain [20]. In rats treated with oxaliplatin (4 mg/kg) twice a week for 4 weeks, the expression of NR2B in spinal cord was found to increase [13]. In addition, the demyelination of peripheral nerves is also involved in neuropathic pain [21]. NRG1 is one of the myelination regulatory factors in peripheral and central nervous systems [22–24]. A recent study showed that treatment with oxaliplatin (4 mg/kg) twice a week for 4 weeks induces demyelination of peripheral nerves in rats and decreases the expression of NRG1 in sciatic nerve [14]. In this study, in mice treated with a single injection of oxaliplatin (3 mg/kg), the expression of NR2B in the spinal cord and of NRG1 in the sciatic nerve were not altered compared to that in control mice. These findings suggest that repeated, but not a single, administration of oxaliplatin affects the expression of NR2B and NRG1. In addition, KSOT did not affect their expression either, suggesting that KSOT inhibits the exacerbation of mechanical allodynia through other mechanisms.

Spinal microglia and astrocytes, especially their reactivation, play an important role in neuropathic pain [15–17, 25–28]. In the present study, a single injection of oxaliplatin increased the number of reactivated astrocytes, but not that of microglia. Robinson et al. [29] also reported the same result in rats. Thus, it is suggested that reactivated astrocytes are involved in oxaliplatin-induced mechanical allodynia. The several cytokines (e.g., IL-1*β*, IL-6, TNF-*α*, IL-18) released from reactivation of microglia contributed to the reactivation of astrocytes [28]. Repetitive administration of oxaliplatin (4 mg/kg ip) twice a week for 4 weeks (total of nine injections) increases the expression of IL-6 and TNF-*α* in the spinal dorsal horn, resulting in reactivation of astrocytes [30]. However, in this study, a single injection of oxaliplatin did not affect the reactivation of microglia. Thus, another pathway may be involved in the reactivation of astrocytes. Oxaliplatin is known to directly activate the primary afferents [31, 32]. Furthermore, the primary afferent-derived transmitters (e.g., glutamate, substance P, calcitonin gene-related peptide, and ATP) directly reactivate the astrocytes [28]. However, in this study, a single administration of KSOT did not inhibit oxaliplatin-induced mechanical allodynia. In addition, oxaliplatin does not release substance P from DRG neurons [33]. Astrocyte-specific gap junction protein connexin 43 (Cx43) is also involved in the reactivation of astrocytes in spinal dorsal horn and the oxaliplatin-induced neuropathic pain [34]. Taken together, KSOT may inhibit the expression of neurotransmitters and Cx43 involved in the reactivation of astrocytes. As the inhibitory mechanisms of the reactivation of astrocytes by KSOT still remain unclear, it might be a fruitful area of research for future studies.

It is reported that spinal astrocytes, but not microglia, contribute to painful neuropathy in rats induced by repeated treatments with chemotherapy drugs (paclitaxel, oxaliplatin, vincristine, and bortezomib) [29, 34–36]. In this study, KSOT inhibited mechanical allodynia in mice treated oxaliplatin, but not paclitaxel, vincristine, and bortezomib. In the present, the mechanisms of the differences of the antiallodynic action of KSOT in mice treated with chemotherapy drugs remain unclear. Our mouse model is developed by a single treatment with chemotherapy drug. However, rat model is developed by repeated treatments with chemotherapy drug. Therefore, in addition to the strain, the method of treatment of chemotherapy drug may be involved in the differences of antiallodynic action of KSOT. Furthermore, the process of astrocyte activation may differ depending on the type of chemotherapy drugs. This may be involved in the action of KSOT. These speculations will be resolved in our future study.

KSOT consists of seven herbal medicines: Cinnamomi ramulus, Zingiberis rhizoma, Zizyphi fructus, Glycyrrhizae radix, Ephedrae herba, Asiasari radix, and Aconiti Tuber. Prophylactic repetitive oral administration of the dried extract of Aconiti Tuber (Bushi) does not inhibit oxaliplatin-induced mechanical allodynia in mice [37]. On the other hand, prophylactic repetitive oral administration of the dried extract of traditional herbal formulations comprising Zingiberis rhizome and Zizyphi fructus (keishikajutsubuto, ogikeishigomotsuto and Gyejigachulbu-tang) [38–40] inhibits oxaliplatin-induced peripheral neuropathy in human patients [38, 39] and mechanical hypersensitivity in rats [40]. Zingiberis rhizome and the active component 6-gingerol inhibit the activation of astrocytes [41, 42]. Oleanolic acid, which is an active component of Zizyphi fructus, and Gyejigachulbu-tang comprising Zizyphi fructus also inhibit the activation of astrocytes [40, 43]. In summary, the components in Zingiberis rhizome and Zizyphi fructus may contribute to the inhibition of reactivation of spinal astrocytes, thus, regulating the exacerbation of oxaliplatin-induced mechanical allodynia. Therefore, current trends suggested that traditional herbal formulations comprising Zingiberis rhizome and Ziziphi fructus may be effective against oxaliplatin-induced peripheral neuropathy.

## 5. Conclusion

Prophylactic repetitive oral administration of KSOT inhibited the exacerbation of oxaliplatin-induced mechanical allodynia. It is suggested that a decrease in the number of spinal reactivated astrocytes by KSOT treatment is partly involved in the antiallodynic mechanisms. These results suggest that remedies against chemotherapy-induced allodynia should be selected depending on the chemotherapeutic agent used.

## Figures and Tables

**Table 1:** The components of KSOT.

herbal medicine	(g)
Cinnamomi cortex	3
Zingiberis rhizoma	1
Ziziphi fructus	3.3
Glycyrrhizae radix	2
Ephedrae herba	2
Asiasari radix	2
Aconiti Tuber	3.5

## Data Availability

The data used in this study is available from the corresponding author upon request.
